# Thrombus containing lesions strategies during primary percutaneous coronary interventions in ST-segment elevation myocardial infarction: insights from ORPKI National Registry

**DOI:** 10.1007/s11239-023-02811-z

**Published:** 2023-04-24

**Authors:** Tomasz Rakowski, Michał Węgiel, Krzysztof P. Malinowski, Zbigniew Siudak, Wojciech Zasada, Barbara Zdzierak, Tomasz Tokarek, Łukasz Rzeszutko, Dariusz Dudek, Stanisław Bartuś, Andrzej Surdacki, Artur Dziewierz

**Affiliations:** 1 2nd Department of Cardiology, Institute of Cardiology, Jagiellonian University Medical College, 2 Jakubowskiego St., 30-688 Kraków, Poland; 2 Clinical Department of Cardiology and Cardiovascular Interventions, University Hospital, Kraków, Poland; 3 Department of Bioinformatics and Telemedicine, Jagiellonian University Medical College, Kraków, Poland; 4 Collegium Medicum, Jan Kochanowski University, Kielce, Poland; 5 Center for Invasive Cardiology, Electrotherapy and Angiology, Nowy Sacz, Poland; 6 Center for Innovative Medical Education, Jagiellonian University Medical College, Kraków, Poland

**Keywords:** Percutaneous coronary intervention, Registries, Glycoprotein IIb/IIIa inhibitors, Aspiration thrombectomy, Myocardial infarction, Antiplatelet therapy

## Abstract

In the era of potent P2Y_12_ inhibitors, according to current guidelines, treatment with glycoprotein IIb/IIIa inhibitors (GPIs) should be limited to bail-out and/or highly thrombotic situations. Similarly, the recommendation for aspiration thrombectomy (AT) is downgraded to very selective use. We examine the prevalence, and predictors of GPI and AT use in STEMI patients referred to primary percutaneous coronary intervention (PCI). Data on 116,873 consecutive STEMI patients referred to primary PCI in Poland between 2015 and 2020 were analyzed. GPIs were administered in 29.3%, AT was used in 11.6%, and combined treatment with both in 6.1%. There was a mild trend toward a decrease in GPI and AT usage during the analyzed years. On the contrary, there was a rapid growth of the ticagrelor/prasugrel usage rate from 6.5 to 48.1%. Occluded infarct-related artery at baseline and no-reflow during PCI were the strongest predictors of GPI administration (OR 2.3; 95% CI 2.22–2.38 and OR 3.47; 95% CI 3.13–3.84, respectively) and combined usage of GPI and AT (OR 4.4; 95% CI 4.08–4.8 and OR 3.49; 95% CI 3.08–3.95 respectively) in a multivariate logistic regression model. Similarly, the administration of ticagrelor/prasugrel was an independent predictor of both adjunctive treatment strategies. In STEMI patients in Poland, GPIs are selectively used in one in four patients during primary PCI, and the combined usage of GPI and AT is marginal. Despite the rapid growth in potent P2Y_12_ inhibitors usage in recent years, GPIs are selectively used at a stable rate during PCI in highly thrombotic lesions.

**Supplementary Information:**

The online version contains supplementary material available at 10.1007/s11239-023-02811-z.

## Highlights

- GPIs and aspiration thrombectomy are selectively used in contemporary STEMI patients during primary PCI in Poland, with GPIs administered in one in four patients and aspiration thrombectomy in one in ten patients.
- The rapid growth in the use of potent P2Y12 inhibitors, such as ticagrelor and prasugrel, in Poland has not led to a significant reduction in the use of GPIs and aspiration thrombectomy during primary PCI in STEMI patients.
- The target population for GPI administration and/or aspiration thrombectomy during primary PCI in STEMI is characterized by a higher ischemic risk profile, indicating that these strategies are being used selectively in patients with a greater need for aggressive treatment.

## Introduction

Rapid platelet inhibition is a crucial part of reperfusion therapy with primary percutaneous coronary interventions (PCI) in patients with ST-elevation myocardial infarction (STEMI). Glycoprotein IIb/IIIa inhibitors (GPIs) were routinely used as adjunctive periprocedural pharmacotherapy in the era of clopidogrel [1, 2]. After introducing novel, potent P2Y_12_ inhibitors, ticagrelor and prasugrel, the role of GPIs remains discussed due to unclear benefits and safety of such combined therapy [3, 4]. According to current guidelines, their usage should be limited to bail-out and/or highly thrombotic situations [5]. GPIs can be either used in adjunct to oral P2Y12 inhibitors in patients pre-treated before PCI or as a bridging strategy in patients in which a P2Y_12_ inhibitor was not administered before PCI [6]. Similarly, the recommendation for the aspiration thrombectomy strategy for thrombotic lesions in STEMI was downgraded in current guidelines to very selective but not routine use [7]. On the other hand, the onset of action of potent oral P2Y_12_ inhibitors may be delayed in STEMI patients, especially in those with hemodynamic instability or concomitant opioid administration [7, 8]. These all make the landscape of the thrombus-containing lesion treatment strategy rather defensive, underlying the need for individual approach to STEMI patients. Data concerning the daily clinical practice of GPIs usage and the combined strategy of GPIs with aspiration thrombectomy are lacking.

Presented study aimed to examine the prevalence, procedural characteristics, and predictors of GPI administration in all-comers contemporary STEMI patients referred to primary PCI in Poland. Considering the procedural approach to thrombus-containing lesions we also analyzed data on the combined usage of GPIs and aspiration thrombectomy.

## Methods

The presented analysis focused on consecutive STEMI patients referred to Primary PCI in Poland between 2015 and 2020. Data were prospectively collected and stored via electronic case report forms in the National PCI Registry (ORPKI) operated by the Jagiellonian University Medical College in Krakow and endorsed by the Polish Association of Cardiovascular Interventions of the Polish Cardiac Society. ORPKI is a national registry collecting data on all PCI procedures performed in Poland [9–11]. For this analysis data on 116,873 consecutive PCI procedures in STEMI were retrieved from the database. Patients’ demography, baseline clinical characteristics, angiography and PCI details, as well as periprocedural pharmacotherapy were analyzed. Information on GPI administration included whether the drug was administered and which one was given (abciximab, eptifibatide, or tirofiban), but did not include details on the method of bolus administration (intravenous or intracoronary) or infusion specifics. Two GPIs administration approaches were described: "adjunctive" therapy in patients pretreated early with P2Y_12_ inhibitors and "bridging" therapy in patients in which a P2Y_12_ inhibitor was not administered before PCI. Angiography was assessed by PCI operators but not by the core-lab. We focused on two strategies during procedures: (1) GPIs administration and (2) the combined usage of GPIs and aspiration thrombectomy. Results were also analyzed in the context of potent P2Y_12_ inhibitors periprocedural treatment (ticagrelor, prasugrel). Additionally, we analyzed the usage of both treatment strategies according to site STEMI volume.

### Statistical analysis

Standard descriptive statistics were used in the analysis. Quantitative variables were described with median with interquartile range (for non-normal distribution of data). Normality was assessed by the Shapiro–Wilk test. Categorical variables were presented as percentages. Chi square test or Fisher Exact Test were used for comparing categorical data, as appropriate. Temporal trends were analyzed with Cochran-Armitage trend test. A logistic regression analysis was constructed to identify predictors of the use of GPIs and aspiration thrombectomy. Variables, including demography, clinical characteristics, and angiography parameters, were considered by calculating simple models. Final multiple models were constructed using a stepwise combined (forward/backward) technique with minimization of the Bayesian Information Criterion as a target, taking into account all variables with p-value from simple model < 0.2 or of clinical importance. Results are presented as odds ratios with an associated 95% confidence interval. The level of statistical significance was set at alpha value < 0.05. All statistical analyses were performed using the R version 4.1.1 (R Foundation for Statistical Computing, Vienna, Austria, 2021) with package “rms” version 6.2–0 and JMP 15.2 (SAS Institute Inc., Cary, NC, USA, 2020).

## Results

**Fig. 1:**
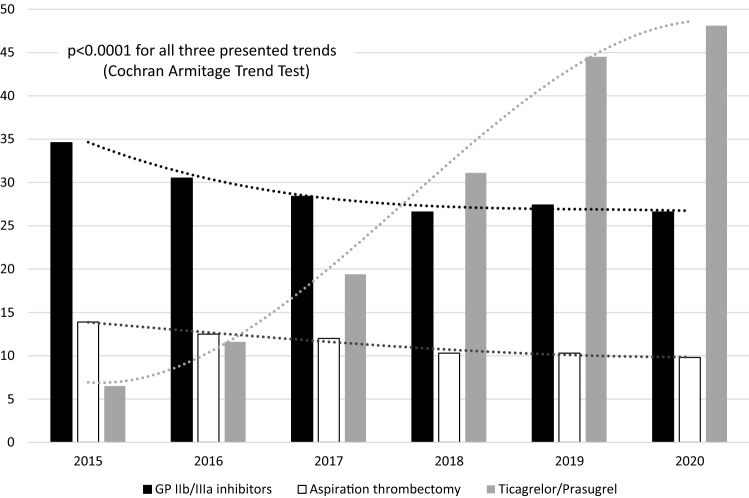
Temporal trends in glycoprotein (GP) IIb/IIIa inhibitors administration (black), aspiration thrombectomy (white) and ticagrelor or prasugrel administration (grey) in consecutive ST-segment elevation myocardial infarction patients referred to primary PCI in Poland between 2015 and 2020 (p < 0.0001 for all three trends)

During six years, 116,873 consecutive patients in STEMI were treated with primary PCI. GPIs were administered in 29.3% of these patients (eptifibatide 23.6%, abciximab 5.3%, tirofiban 0.4%), aspiration thrombectomy was used in 11.6%, and combined treatment with GPIs and aspiration thrombectomy in 6.1%. There was a trend towards decrease in GPIs and aspiration thrombectomy usage during the analyzed years, which was mainly seen between 2015 to 2018 with a plateau beyond 2018 at the level of about 27% for GPIs and about 10% for aspiration thrombectomy. Conversely, there was rapid growth in ticagrelor/prasugrel usage rate from 6.5% in 2015 to 48.1% in 2020 (Fig. 1).

### GP IIb/IIIa inhibitors administration

Patients with periprocedural GPIs administration were younger, more often men, with history of previous PCI and smoking, and presented with cardiogenic shock on admission. They were less likely to have diabetes, chronic kidney disease and previous stroke (Table 1). In the GPIs group, we observed a higher rate of occluded infarct-related artery at baseline (more often left main or/and left anterior descending artery), PCI of bifurcation, no-reflow during PCI, use of aspiration thrombectomy and administration of ticagrelor/prasugrel (Table 1). Of the 34,208 GPIs treated patients, those drugs were administered as "adjunctive" therapy in 59.1% of patients or as "bridging" therapy in 40.9% of patients. When analyzed patients pretreated with P2Y_12_ inhibitors before PCI, GPIs were administered in 31% of those patients.

**Table 1:** Demography, clinical and procedural characteristics according to glycoprotein IIb/IIIa inhibitors administration

	Glycoprotein IIb/IIIa inhibitor (+) n = 34,208 (29.3%)	Glycoprotein IIb/IIIa inhibitor (−) n = 82,665 (70.7%)	Total n = 116,873	P
Age (years) (Q1; Q3)	63 (56; 70)	66 (58; 75)	65 (58; 73)	< 0.001
Male gender (%)	71.9	66.4	68.1	< 0.001
Arterial hypertension (%)	58.8	58.2	58.4	0.07
Diabetes mellitus (%)	16.7	17.8	17.5	< 0.001
Chronic kidney disease (%)	2.6	3.7	3.4	< 0.001
Smoking (%)	34.1	28.7	30.3	< 0.001
COPD (%)	2.1	2.1	2.1	0.63
Previous MI (%)	12.5	12.4	12.5	0.61
Previous PCI (%)	12.4	11.7	11.9	< 0.001
Previous CABG (%)	1.7	1.8	1.7	0.33
Previous stroke (%)	2.4	3.5	3.2	< 0.001
Killip class on admission:				< 0.001
I (%)	79.0	84.6	82.9	
II (%)	11.9	9.4	10.1	
III (%)	4.2	2.7	3.2	
IV (%)	4.9	3.3	3.8	
Cardiac arrest before admission (%)	5.3	4.8	4.9	0.0016
Femoral access (%)	23.8	22.3	22.7	< 0.001
Angiography result				< 0.001
Single vessel (%)	45.6	45.3	45.4	
Multivessel without LMCA (%)	46.8	47.9	47.6	
Multivessel with LMCA (%)	7.2	6.6	6.8	
LMCA only (%)	0.4	0.2	0.2	
Infarct related artery				< 0.001
LMCA (%)	3.2	2.1	2.5	
LAD (%)	43	40.2	41	
Cx (%)	13.0	14.0	13.7	
RCA (%)	41.4	38.9	39.6	
Other (%)		4.8	3.2	
Bifurcation (%)	8.6	7.0	7.5	< 0.001
Aspiration thrombectomy (%)	20.9	7.8	11.6	< 0.001
P2Y_12_ inhibitors				< 0.001
Clopidogrel (%)	59.9	64.7	63.3	
Prasugrel (%)	3.3	1.9	2.3	
Ticagrelor (%)	36.8	33.4	34.4	
Administration before PCI (%)	59.1	58.8	58.9	0.31
Administration during PCI (%)	40.9	41.2	41.1	
TIMI flow before PCI				< 0.001
0 (%)	72.6	52	58.1	
1 (%)	11.7	16	14.8	
2 (%)	9.6	16.8	14.7	
3 (%)	6.1	15.2	12.4	
TIMI flow after PCI				< 0.001
0 (%)	1.8	2.5	2.3	
1 (%)	2	1.4	1.5	
2 (%)	6.8	3.9	4.8	
3 (%)	89.4	92.2	91.4	
Stent implantation (%)	92.4	91.4	91.7	< 0.001
Drug eluting stents (%)	89.8	88.7	89.1	< 0.001
Total amount of contrast (ml) (Q1: Q3)	160 (130; 200)	150 (120; 200)	150 (120; 200)	< 0.001
Total radiation dose (mGy) (Q1; Q3)	796 (451; 1356)	676 (390; 1144)	707 (405; 1206)	< 0.001
Time from pain onset to PCI (min) (Q1; Q3)	230 (136; 480)	245 (147;540)	240 (143; 522)	< 0.001
No-reflow (%)	3.2	0.8	1.5	< 0.001
Cardiac arrest during procedure (%)	2.5	1.4	1.8	< 0.001

*COPD* chronic obstructive pulmonary disease, *MI* myocardial infarction, *PCI* percutaneous coronary intervention, *CABG* coronary artery bypass grafting, *LMCA* left main coronary artery, *LAD* left anterior descending artery, *Cx* circumflex artery, *RCA* right coronary artery, *TIMI* thrombolysis in myocardial infarction

In a multivariate logistic regression model, the occluded infarct-related artery at baseline and no-reflow during PCI were the strongest independent predictors of GPIs administration. Others positive predictors were male sex, smoking, cardiogenic shock on admission, the culprit within left main and/or left anterior descending coronary artery, previous PCI, bifurcation treatment, administration of ticagrelor/prasugrel, and aspiration thrombectomy usage. The negative predictors were age, chronic kidney disease, previous stroke, and cardiac arrest before admission. Results multivariate logistic regression analysis are presented in Table 2. Results of univariate logistic regression are presented in table S1 in supplementary information.

**Table 2:** Multivariable logistic regression analyses for predicting glycoprotein IIb/IIIa inhibitors administration

	OR	95% CI	*P* value
Age (per 1 year)	0.98	0.98–0.98	< 0.001
Sex (male)	1.12	1.09–1.16	< 0.001
Previous stroke	0.71	0.65–0.77	< 0.001
Previous PCI	1.12	1.08–1.17	< 0.001
Smoking	1.07	1.04–1.1	< 0.001
Chronic kidney disease	0.78	0.72–0.85	< 0.001
Killip class IV on admission	1.58	1.47–1.7	< 0.001
Ticagrelor or prasugrel	1.08	1.05–1.11	< 0.001
Cardiac arrest before admission	0.89	0.84–0.95	< 0.001
Aspiration thrombectomy during PCI	2.58	2.49–2.68	< 0.001
TIMI 0 or 1 in baseline angiography	2.3	2.22–2.38	< 0.001
IRA in LMCA and/or LAD	1.2	1.17–1.23	< 0.001
PCI of bifurcation	1.24	1.18–1.3	< 0.001
No-reflow during PCI	3.47	3.13–3.84	< 0.001

*PCI* percutaneous coronary intervention, *IRA* infarct-related artery, *LMCA* left main coronary artery, *LAD* left anterior descending artery, *TIMI* thrombolysis in myocardial infarction;

### Combined usage of GP IIb/IIIa inhibitors and aspiration thrombectomy

Patients with the combined strategy with periprocedural GPIs administration and aspiration thrombectomy were younger, more often men, with a history of smoking, and presented with cardiogenic shock on admission. They were less likely to have diabetes mellitus, chronic kidney disease, and previous stroke (Table S2 in supplementary information). In angiography, we observed a higher rate of the occluded infarct-related artery in baseline angiography (more often the right coronary artery) and a higher rate of no-reflow during PCI. They more often received ticagrelor/prasugrel (Table S2 in supplementary information).

Similarly to GPIs administration analysis, the occluded infarct-related artery at baseline and no-reflow during PCI were the strongest independent predictors of combined usage of GP IIb/IIIa inhibitors and aspiration thrombectomy in a multivariate logistic regression model. Results of univariate and multivariate logistic regression analysis are presented in table S3 in supplementary information.

### Results according to site STEMI volume

**Fig. 2:**
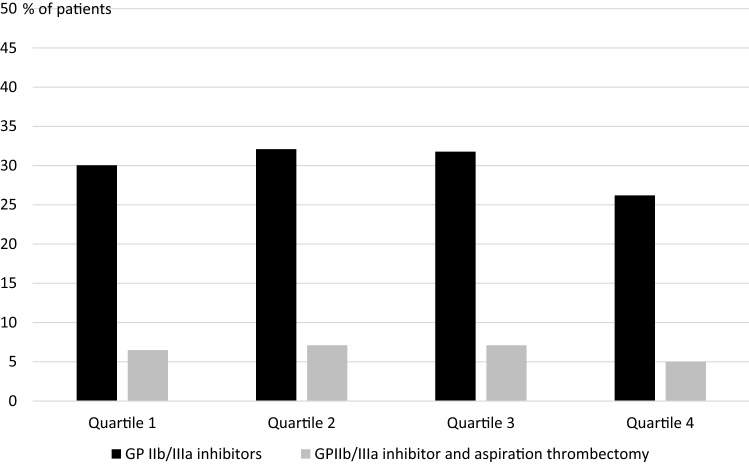
Glycoprotein (GP) IIb/IIIa inhibitors administration (black) and combined strategy (GP IIb/IIIa inhibitors administration and aspiration thrombectomy) (grey) according to site ST-segment elevation myocardial infarction volume (quartiles)

We analyzed the usage of both treatment strategies (rate of GPIs administration and combined treatment with of GPIs and aspiration thrombectomy) according to site STEMI volume (quartiles). Data according to site volume (primary PCI in STEMI) quartiles are presented in Fig. 2. No relationship between treatment strategies and site volume was confirmed.

## Discussion

The most important findings from the presented analysis are: (1) in all-comers contemporary STEMI patients in Poland, GPIs and aspiration thrombectomy are selectively used in respectively one in four and one in ten patients during primary PCI in STEMI, (2) the combined treatment strategy with both GPIs and aspiration thrombectomy during primary PCI is marginal, (3) the use of those strategies was not particularly reduced by the rapid growth in potent P2Y12 inhibitors use in Poland in recent years, (4) target STEMI population for GPI administration is characterized by high ischemic risk.

Primary PCI of thrombotic lesions in patients with STEMI may be challenging due to the risk of no-reflow and distal embolization. Rapid platelet inhibition plays an important role during mechanical reperfusion [12, 13]. GPIs were routinely used as adjunctive periprocedural pharmacotherapy during primary PCI for many years [1]. Several reports have confirmed their beneficial effect in patients with STEMI [14, 15]. Worth noticing is that most older trials concerning GPIs were performed with clopidogrel. Nowadays, in the era of more potent P2Y_12_ inhibitors, ticagrelor and prasugrel, the role of GPIs is debatable mainly due to the lack of clear data on the benefits and bleedings risks of such an approach. According to current guidelines, their usage should be limited to bail-out and/or highly thrombotic situations [5]. These may influence everyday clinical practice and lower the rate of GPI usage, especially with high penetration of potent P2Y_12_ inhibitors. Over, the results of our analysis do not fully confirm these assumptions, as we observed only a mild trend towards decrease in use of GPIs through years 2015–2020, despite the pronounced increase in use of ticagrelor and prasugrel. Blanchart K et al. showed (based on prospective registry data) the GPI usage in 41% of STEMI patients treated with ticagrelor or prasugrel during primary PCI. Importantly, 52% of those patients have received GPI as a systematic strategy [16]. Similarly, in the analysis of the FAST-MI program, GPIs were used in about 40% of STEMI patients during PCI, with 54% of ticagrelor or prasugrel administration (including prehospital dual antiplatelet therapy) [17]. The main concern about treatment with GPI on top of potent P2Y_12_ inhibitors is elevated bleeding risk. However, to date, there is no dedicated clinical trial to assess such risk (and potential clinical benefit) in the contemporary STEMI cohort. In the above-mentioned registry with the 98% of radial approach there was no significant increase of bleeding in the GPI cohort [16]. Meta-analysis of PLATO, TRITON-TIMI 38, and CURRENT-OASIS 7 trials have shown a similar risk of bleedings with GPI and potent P2Y_12_ inhibitors compared to GPI and clopidogrel. The increase in bleeding risk with GPIs was present regardless of the type of P2Y_12_ inhibitor but relative risk was higher with clopidogrel than with ticagrelor or prasugrel. This suggests that GPIs treatment is the main factor of elevated bleeding risk, and administration of potent P2Y_12_ inhibitors may have no further influence on bleeding risk [18]. On the other hand, there is no clear evidence of the clinical benefit of GPIs administration on top of prasugrel and ticagrelor, and the benefit of novel P2Y_12_ inhibitors compared to clopidogrel in reducing the risk of ischemic events is independent of GPI use [16]. This underlines the role of the individual, tailored approach to identify patients who benefit from GPIs administration the most. According to our analysis, GPIs are administered in patients with the higher ischemic risk profile and relatively lower bleeding risk profile, with occluded infarct-related artery and/or as bail-out for no-reflow. Another possible approach selectively used in some centers is intracoronary GPI bolus not followed by infusion. The idea of this strategy is based on the benefit of rapid antiplatelet effect in STEMI without aggressive prolonged treatment (earlier offset of action) as a potential balanced approach to optimize bleeding and ischemic risks. Galli M et al. showed such approach as safe in selected patients at high ischemic risk both in those pretreated and in those naïve to P2Y_12_ inhibitors. [6]. In our study, GPIs were administered as "adjunctive" therapy to approximately 60% of patients receiving GPIs, a practice largely driven by the prevalent clinical strategy of initiating early dual antiplatelet therapy in STEMI patients before PCI. In those early treated with P2Y_12_ inhibitor, GPIs were administered in about one third what is slightly lower rate comparing to other studies [16, 17].

Aspiration thrombectomy is a tool designed for mechanical thrombus removal and to prevent distal embolization [19]. In initial reports, a beneficial effect of routine thrombectomy on angiographic and short-term clinical outcomes in STEMI patients was observed [20–23]. Those relatively small-scale studies were performed in centers with high experience in aspiration thrombectomy technique. However, further large-scale trials with routine aspiration thrombectomy in STEMI did not confirm any clinical benefit [24–26]. Moreover, the question was raised about the safety of routine use of thrombectomy before PCI taking into account the increased risk of stroke [27, 28]. Potential benefit of aspiration thrombectomy is multifactorial including optimal patients selection and adequate technique. Similarly to GPIs, recent recommendations advocate against the routine use of thrombectomy (class III for routine usage) [5]. In our registry, we observed only a mild trend toward decreased use of thrombectomy throughout the analyzed years. On the other hand, the combined use of both GPIs and thrombectomy applied to a small proportion of patients. It may be driven by guidelines and class III recommendation, which may be understood as contraindications but rather should be as very selective usage. In the registry data presented by Blanchart K et al. thrombectomy was used in 26% of patients, mostly as adjunctive therapy to GPIs [16]. In our analysis, no-reflow phenomenon and occluded infarct-related artery at baseline were the strongest predictors for this kind of pharmaco-mechanical approach to thrombus lesion and similarly to GPIs alone it was mainly used in patients with the higher ischemic risk profile. Some novel thrombectomy techniques are under evaluation to improve treatment results [29].

The presented study has several limitations. First, the observation is limited only to procedure time, and no longer follow-up was available. Analysis is based on registry data describing everyday clinical practice; thus, it is impossible to compare the outcomes of different strategies with GPIs and aspiration thrombectomy due to important differences in baseline characteristics. Because of the two above-mentioned limitations, there is no point in performing further statistical analysis for in-cathlab outcomes, even including propensity score matching. Data about the way of bolus administration (intravenous or intracoronary) and infusion details are unavailable. However, routine practice is to give infusion after bolus injection. Finally, angiograms were assessed by operators. No core lab analysis was performed in this registry.

## Conclusions

The presented analysis shows that GPIs are used in primary PCI in STEMI in one in four patients in Poland, and combined use of GPIs and aspiration thrombectomy is marginal. An increase in ticagrelor and prasugrel use does not seem to impact rates of use of GPIs and thrombectomy in Poland. The target population for GPI and/or thrombectomy is characterized by high ischemic risk.

## Supplementary Information

Below is the link to the electronic supplementary material.

Supplementary file1 (DOCX 24 kb)

## Data Availability

The data that support the findings of this study are available from the corresponding author upon reasonable request.
