# Supplementary material for: Thrombus containing lesions strategies during primary percutaneous coronary interventions in ST-segment elevation myocardial infarction: insights from ORPKI National Registry
Source: J Thromb Thrombolysis. 2023 Apr 24;56(1):156–63. doi: 10.1007/s11239-023-02811-z (PMC10284931; doi:10.1007/s11239-023-02811-z)
Supplement: Supplementary file 1 — Supplementary file1 (DOCX 24 kb) [file 11239_2023_2811_MOESM1_ESM.docx]

**Supplementary information**

Table S1. Univariable logistic regression analyses for predicting glycoprotein IIb/IIIa inhibitors administration.

|  | **OR** | **95%CI** | ***P* value** |
| --- | --- | --- | --- |
| Age (per 1 year) | 0.979 | 0.978 – 0.98 | <0.001 |
| Sex (male) | 1.29 | 1.26 - 1.33 | <0.001 |
| Diabetes mellitus | 0.93 | 0.9 - 0.97 | <0.001 |
| Previous stroke | 0.66 | 0.61 – 0.72 | <0.001 |
| Previous MI | 1.01 | 0.97 – 1.05 | 0.61 |
| Previous PCI | 1.07 | 1.03 – 1.1 | <0.001 |
| Previous CABG | 0.95 | 0.86 – 1.05 | 0.32 |
| Smoking | 1.28 | 1.25 – 1.32 | <0.001 |
| Arterial hypertension | 1.02 | 1.0 – 1.05 | 0.068 |
| Chronic kidney disease | 0.71 | 0.66 – 0.77 | <0.001 |
| COPD | 0.98 | 0.9 – 1.07 | 0.63 |
| Killip class IV on admission | 1.54 | 1.44 – 1.65 | <0.001 |
| Ticagrelor or prasugrel | 1.23 | 1.19 – 1.26 | <0.001 |
| Cardiac arrest before admission | 1.1 | 1.04 – 1.16 | 0.001 |
| Aspiration thrombectomy during PCI | 3.14 | 3.02 – 3.25 | <0.001 |
| TIMI 0 or 1 in baseline angiography | 2.53 | 2.45 – 2.62 | <0.001 |
| IRA in LMCA and/or LAD | 1.14 | 1.11 – 1.17 | <0.001 |
| PCI of bifurcation | 1.25 | 1.2 – 1.31 | <0.001 |
| No-reflow during PCI | 3.85 | 3.5 – 4.3 | <0.001 |

COPD, chronic obstructive pulmonary disease; MI, myocardial infarction; PCI, percutaneous coronary intervention; CABG, coronary artery bypass grafting; IRA, infarct-related artery; LMCA, left main coronary artery; LAD, left anterior descending artery; TIMI, Thrombolysis in myocardial infarction;

Table S2. Demography, clinical and procedural characteristics according to combined usage of glycoprotein IIb/IIIa inhibitors and aspiration thrombectomy.

|  | **Glycoprotein IIb/IIIa inhibitor and aspiration thrombectomy (+)**  **n=7161 (6.1%)** | **Glycoprotein IIb/IIIa inhibitor and aspiration thrombectomy (-)**  **n=109712 (93.9%)** | **Total**  **n=116873** | **P** |
| --- | --- | --- | --- | --- |
| Age (years) (Q1; Q3) | 62 (55; 69) | 65 (58; 74) | 65 (58; 73) | <0.001 |
| Male gender (%) | 72.8 | 67.7 | 68.1 | <0.001 |
| Arterial hypertension (%) | 58.8 | 58.4 | 58.4 | 0.44 |
| Diabetes mellitus (%) | 16.2 | 17.6 | 17.5 | 0.002 |
| Chronic kidney disease (%) | 3.0 | 3.4 | 3.4 | 0.05 |
| Smoking (%) | 38.3 | 29.8 | 30.3 | <0.001 |
| COPD (%) | 2.0 | 2.1 | 2.1 | 0.52 |
| Previous MI (%) | 12.7 | 12.4 | 12.5 | 0.58 |
| Previous PCI (%) | 12.5 | 11.8 | 11.8 | 0.076 |
| Previous CABG (%) | 1.7 | 1.7 | 1.7 | 0.67 |
| Previous stroke (%) | 2.4 | 3.2 | 3.2 | <0.001 |
| Killip class on admission:  I (%)  II (%)  III (%)  IV (%) | 77.4  10.9  5.8  5.9 | 83.3  10.1  3.0  3.6 | 82.9  10.1  3.2  3.8 | <0.001 |
| Cardiac arrest before admission (%) | 6 | 4.9 | 4.9 | <0.001 |
| Femoral access (%) | 21.3 | 23.6 | 23.5 | <0.001 |
| Angiography result:  Single vessel (%)  Multivessel without LMCA (%)  Multivessel with LMCA (%)  LMCA only (%) | 49.3  45.0  5.3  0.4 | 45.1  47.8  6.9  0.2 | 45.4  47.6  6.8  0.2 | <0.001 |
| Infarct related artery:  LMCA (%)  LAD (%)  Cx (%)  RCA (%)  Other (%) | 2.1  39.4  9.5  49.1 | 2.5  41.1  14.0  39.0  3.4 | 2.5  41.0  13.7  39.6  3.2 | <0.001 |
| Bifurcation (%) | 8.8 | 7.4 | 7.5 | <0.001 |
| Aspiration thrombectomy (%) | 100 | 5.86 | 11.6 | <0.001 |
| P2Y_12_ inhibitors:  Clopidogrel (%)  Prasugrel (%)  Ticagrelor (%) | 58.1  2.1  39.8 | 63.6  2.4  34.0 | 63.3  2.3  34.4 | <0.001 |
| TIMI flow before PCI:  0 (%)  1 (%)  2 (%)  3 (%) | 84.6  7.1  5.5  2.8 | 56.3  15.3  15.3  13.1 | 58.1  14.8  14.7  12.4 | <0.001 |
| TIMI flow after PCI:  0 (%)  1 (%)  2 (%)  3 (%) | 2.0  2.2  8.0  87.8 | 2.3  1.5  4.6  91.6 | 2.3  1.5  4.8  91.4 | <0.001 |
| Stent implantation (%) | 91 | 92 | 92 | 0.017 |
| Drug eluting stents (%) | 88.1 | 89.1% | 89.1 | 0.01 |
| Total amount of contrast (ml) (Q1: Q3) | 160 (130; 200) | 150 (120; 200) | 150 (120; 200) | <0.001 |
| Total radiation dose (mGy) (Q1; Q3) | 832 (474; 1398) | 700 (401; 1196) | 707 (405; 1206) | <0.001 |
| No-reflow (%) | 4.5 | 1.3 | 1.5 | <0.001 |
| Cardiac arrest during procedure (%) | 3.4 | 1.7 | 1.8 | <0.001 |

COPD, chronic obstructive pulmonary disease; MI, myocardial infarction; PCI, percutaneous coronary intervention; CABG, coronary artery bypass grafting; LMCA, left main coronary artery; LAD, left anterior descending artery; Cx, circumflex artery; RCA, right coronary artery; TIMI, Thrombolysis in myocardial infarction;

Table S3. Logistic regression analyses for predicting combined usage of glycoprotein IIb/IIIa inhibitors and aspiration thrombectomy.

|  | **OR** | **95%CI** | ***P* value** |
| --- | --- | --- | --- |
| **Univariable regression - glycoprotein IIb/IIIa inhibitor and aspiration thrombectomy** | | | |
| Age (per 1 year) | 0.979 | 0.977 – 0.981 | <0.001 |
| Sex (male) | 1.27 | 1.21 – 1.35 | <0.001 |
| Diabetes mellitus | 0.91 | 0.85 – 0.96 | 0.002 |
| Previous stroke | 0.75 | 0.64 – 0.87 | <0.001 |
| Previous MI | 1.02 | 0.95 – 1.1 | 0.58 |
| Previous PCI | 1.07 | 0.99 – 1.15 | 0.07 |
| Previous CABG | 0.96 | 0.8 – 1.16 | 0.67 |
| Smoking | 1.46 | 1.39 – 1.54 | <0.001 |
| Arterial hypertension | 1.02 | 0.97 – 1.07 | 0.44 |
| Chronic kidney disease | 0.87 | 0.75 – 1.0 | 0.051 |
| COPD | 0.95 | 0.8 – 1.12 | 0.52 |
| Killip class IV on admission | 1.66 | 1.49 – 1.85 | <0.001 |
| Ticagrelor or prasugrel | 1.33 | 1.26 – 1.4 | <0.001 |
| Cardiac arrest before admission | 1.24 | 1.12 – 1.37 | <0.001 |
| TIMI 0 or 1 in baseline angiography | 4.4 | 4.04 – 4.8 | <0.001 |
| IRA in LMCA and/or LAD | 0.92 | 0.88 – 0.97 | <0.001 |
| PCI of bifurcation | 1.21 | 1.11 – 1.32 | <0.001 |
| No-reflow during PCI | 3.49 | 3.08 – 3.95 | <0.001 |
| **Multivariable regression - glycoprotein IIb/IIIa inhibitor and aspiration thrombectomy** | | | |
| Age (per 1 year) | 0.981 | 0.98 – 0.983 | <0.001 |
| Smoking | 1.26 | 1.2 – 1.33 | <0.001 |
| Killip class IV (on admission) | 1.59 | 1.42 – 1.78 | <0.001 |
| Ticagrelor or prasugrel | 1.19 | 1.13 – 1.26 | <0.001 |
| TIMI 0 or 1 in baseline angiography | 4.28 | 3.93 – 4.67 | <0.001 |
| No-reflow during PCI | 3.15 | 2.77 – 3.58 | <0.001 |

COPD, chronic obstructive pulmonary disease; MI, myocardial infarction; PCI, percutaneous coronary intervention; CABG, coronary artery bypass grafting; IRA, infarct-related artery; LMCA, left main coronary artery; LAD, left anterior descending artery; TIMI, Thrombolysis in myocardial infarction;
